# Morphological Features Extracted by AI Associated with Spatial Transcriptomics in Prostate Cancer

**DOI:** 10.3390/cancers13194837

**Published:** 2021-09-28

**Authors:** Eduard Chelebian, Christophe Avenel, Kimmo Kartasalo, Maja Marklund, Anna Tanoglidi, Tuomas Mirtti, Richard Colling, Andrew Erickson, Alastair D. Lamb, Joakim Lundeberg, Carolina Wählby

**Affiliations:** 1 Science for Life Laboratory, Department of Information Technology, Uppsala University, 752 37 Uppsala, Sweden; christophe.avenel@it.uu.se; 2 Department of Medical Epidemiology and Biostatistics, Karolinska Institute, 171 77 Stockholm, Sweden; kimmo.kartasalo@ki.se; 3 Science for Life Laboratory, Department of Gene Technology, KTH Royal Institute of Technology, 171 65 Solna, Sweden; maja.marklund@scilifelab.se (M.M.); joakim.lundeberg@scilifelab.se (J.L.); 4 Department of Clinical Pathology, Uppsala University Hospital, 752 37 Uppsala, Sweden; annatano@yahoo.gr; 5 Department of Pathology, Research Program in Systems Oncology, University of Helsinki, Helsinki University Hospital, 00100 Helsinki, Finland; tuomas.mirtti@helsinki.fi; 6 Nuffield Department of Surgical Sciences, University of Oxford, Oxford OX3 7DQ, UK; richard.colling@nds.ox.ac.uk (R.C.); andrew.erickson@nds.ox.ac.uk (A.E.); alastair.lamb@nds.ox.ac.uk (A.D.L.); 7 Department of Cellular Pathology, Oxford University Hospitals NHS Foundation Trust, Oxford OX3 9DU, UK; 8 Department of Urology, Oxford University Hospitals NHS Foundation Trust, Oxford OX3 7LE, UK

**Keywords:** prostate cancer, morphological features, spatial transcriptomics, deep learning

## Abstract

**Simple Summary:**

Prostate cancer has very varied appearances when examined under the microscope, and it is difficult to distinguish clinically significant cancer from indolent disease. In this study, we use computer analyses inspired by neurons, so-called ‘neural networks’, to gain new insights into the connection between how tissue looks and underlying genes which program the function of prostate cells. Neural networks are ‘trained’ to carry out specific tasks, and training requires large numbers of training examples. Here, we show that a network pre-trained on different data can still identify biologically meaningful regions, without the need for additional training. The neural network interpretations matched independent manual assessment by human pathologists, and even resulted in more refined interpretation when considering the relationship with the underlying genes. This is a new way to automatically detect prostate cancer and its genetic characteristics without the need for human supervision, which means it could possibly help in making better treatment decisions.

**Abstract:**

Prostate cancer is a common cancer type in men, yet some of its traits are still under-explored. One reason for this is high molecular and morphological heterogeneity. The purpose of this study was to develop a method to gain new insights into the connection between morphological changes and underlying molecular patterns. We used artificial intelligence (AI) to analyze the morphology of seven hematoxylin and eosin (H&E)-stained prostatectomy slides from a patient with multi-focal prostate cancer. We also paired the slides with spatially resolved expression for thousands of genes obtained by a novel spatial transcriptomics (ST) technique. As both spaces are highly dimensional, we focused on dimensionality reduction before seeking associations between them. Consequently, we extracted morphological features from H&E images using an ensemble of pre-trained convolutional neural networks and proposed a workflow for dimensionality reduction. To summarize the ST data into genetic profiles, we used a previously proposed factor analysis. We found that the regions were automatically defined, outlined by unsupervised clustering, associated with independent manual annotations, in some cases, finding further relevant subdivisions. The morphological patterns were also correlated with molecular profiles and could predict the spatial variation of individual genes. This novel approach enables flexible unsupervised studies relating morphological and genetic heterogeneity using AI to be carried out.

## 1. Introduction

Prostate cancer is the second most commonly diagnosed cancer and the fifth most common cause of cancer death in men worldwide [1]. Although its mortality rate is on a decreasing trend, prognostication and treatment selection is complicated, largely due to its high genetic and morphological heterogeneity [2]. The relationship between genetic and morphological heterogeneity has been previously suggested, and gene expression differences relate to clinical traits of prostate cancer [3]. Furthermore, higher Gleason grades [4], which evaluate prostate morphology in hematoxylin and eosin (H&E) slides, have been shown to correlate with an increasing number of genetic alterations [5]. Additionally, the current prostate grading consensus proposed by the International Society of Urological Pathology (ISUP) is starting to consider genetic information [6]. Advances in spatially resolved transcriptomics enable spatial profiling of gene expression [7], permitting the simultaneous study of tissue histology and transcriptomics. Recently, Berglund et al. used factor analysis on spatial transcriptomics (ST) data to define transcriptomics profiles that identified diseased prostate areas [8]. The spatial landscape of clonal somatic mutations in benign and malignant tissue is further explored in [9], relating ST data to manually annotated tissue regions. However, to the best of our knowledge, studies aiming to directly correlate automatically extracted quantitative representations of morphology given by histology to transcriptomics signature are lacking for prostate cancer, which may be precisely due to its high heterogeneity.

This challenge has been addressed for other types of cancer through the use of computational methods and artificial intelligence (AI). Classical machine learning approaches included training a support vector machine with ST breast cancer datasets to identify cancer regions [10]. Deep convolutional neural networks have shown exceptional performance for morphological analysis on whole slide images [11], and several tools have been developed to identify cell types combining imaging and ST data [12,13]. Moreover, convolutional neural networks (CNNs) have been used to predict spatial gene expression from histopathology images, achieving reasonable correlation between predicted and true maps [14,15]. Interestingly, He et al. found that the networks learned additional representations that were able to separate malignant and normal tissue [14]. Nonetheless, CNNs trained from scratch require large amounts of training data, and performance is sensitive to variations in staining and imaging setups. Thus, all previous attempts have relied on either networks trained on the lab’s own data endangering generalizability or networks pre-trained with non-histology data, compromising performance.

Thus, we hypothesize that morphological features of prostatectomy H&E sections extracted automatically using a CNN previously trained on prostate needle biopsies could identify histologically and genetically relevant sub-regions inside the tissue in an unsupervised manner. We base our study on the data produced and presented in [9]. It is important to note that we search for correlations between two very large feature spaces: that of the tissue morphology, and that of the spatially resolved gene expression. Furthermore, our dataset is comparably limited. We therefore decided to avoid any kind of over-fitting by handling the two data modalities separately, reducing the morphological feature space to a small number of morphological regions by clustering, and thereafter correlating these regions with gene expression factors and manual tissue annotations. The resulting connections continue to prove the tight correlation between morphology and gene expression, suggesting that neural networks could detect repeated morphological patterns not obvious to the human eye.

## 2. Results

### 2.1. Prostate Cancer Sections and Spatially Resolved Gene Expression

**Figure 1:**
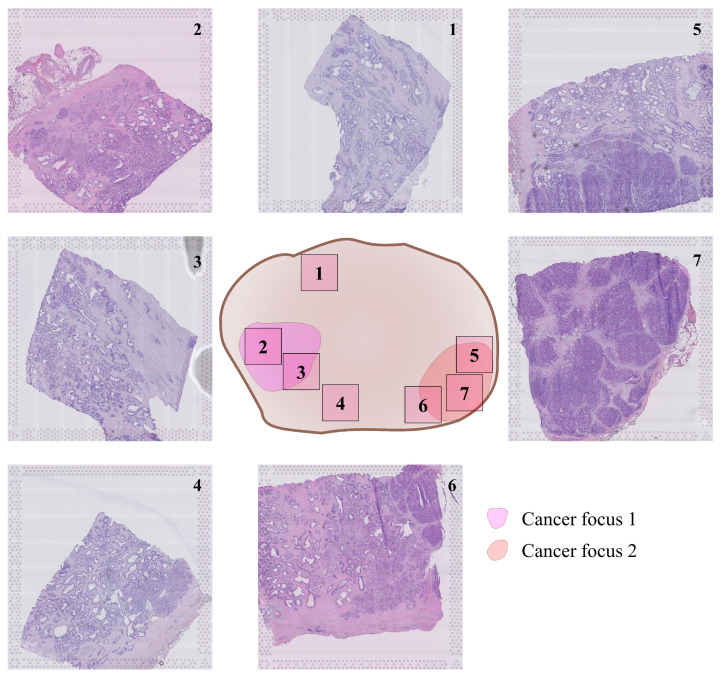
Location of tissue sections inside prostatectomy, provided by Erickson et al. [9].

After radical prostatectomy in a patient with adenocarcinoma, seven H&E-stained tissue sections were selected around the two main tumor foci in the prostate, as shown in Figure 1. ST data were obtained using high-density Visium spatially barcoded spots (10× Genomics, Pleasanton, CA, USA). This yielded a total of 23,282 spots for the combined slides. All details on data collection, factor analysis, and morphological annotations, including ethical considerations, are presented in [9].

### 2.2. Penultimate Layer Activations Are Robust on New Datasets

We started from a pre-trained Gleason grading ensemble CNN developed by Ström et al. [16]. The ensemble was trained on prostate needle biopsies, not on prostatectomy slides, and as is pointed out by Ström et al., these models are sensitive to changes in staining and scanning procedures. Furthermore, the ensemble CNN was designed to perform classification into benign and Gleason patterns 3, 4, and 5, and not to explore organ-wide heterogeneity including, for instance, stroma or intra-tumoral variance. We repurposed the model by extracting the penultimate layer activation from each network to obtain state-of-the-art morphological features that could capture prostate heterogeneity in prostatectomy slides. We made the assumption that for each CNN in the ensemble, the penultimate network layer, which is the last layer before the classification layer of the original network, contains features that describe variations in prostate morphology, which also is true if applied to samples obtained at varying staining and scanning procedures.

**Figure 2:**
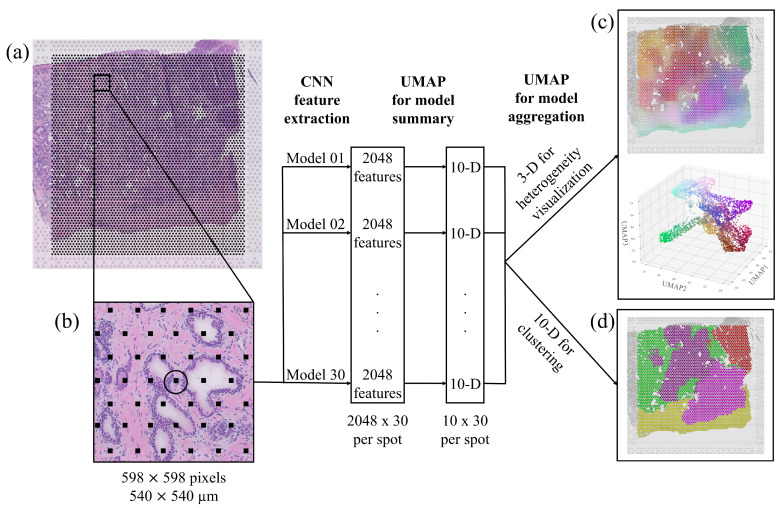
Workflow for deep morphological feature extraction. (**a**) Original section on the whole-slide image. (**b**) A patch of 598×598 pixels extracted centered around an ST spot and fed to the network. The network consists of 30 models, each extracting 2048 features, which are independently reduced to 10 dimensions. Dimensionality reduction is applied again to aggregate the model into (**c**) 3 dimensions where the morphological descriptors are mapped to the original tissue position and presented in a 3D RGB color space, visualizing tissue heterogeneity, and into (**d**) 10 dimensions to cluster tissue regions.

The resulting workflow is presented in Figure 2. The model was designed for input images of size 598×598 pixels at 10× resolution, which equate to around 540×540 μm. Accordingly, we divided the whole slide images into more than 20,000 such patches centered on the ST spot coordinates so that each spot could be associated with an image patch and a set of morphological features. As shown in Figure 2b, each patch covered several ST spots, and neighboring patches overlapped, partially capturing the same tissue morphology. We fed the patches into each of the 30 models and extracted the penultimate layer for each patch and model, and used UMAP dimensionality reduction individually on every model. This reduced the feature space describing the morphology associated with each ST spot to 10×30 dimensions (i.e., a feature vector of length 300). Empirically, we saw that more than 10 features started to yield redundant maps (Supplementary Figure S8).

### 2.3. Deep Morphological Features Identify Tissue Heterogeneity

**Figure 3:**
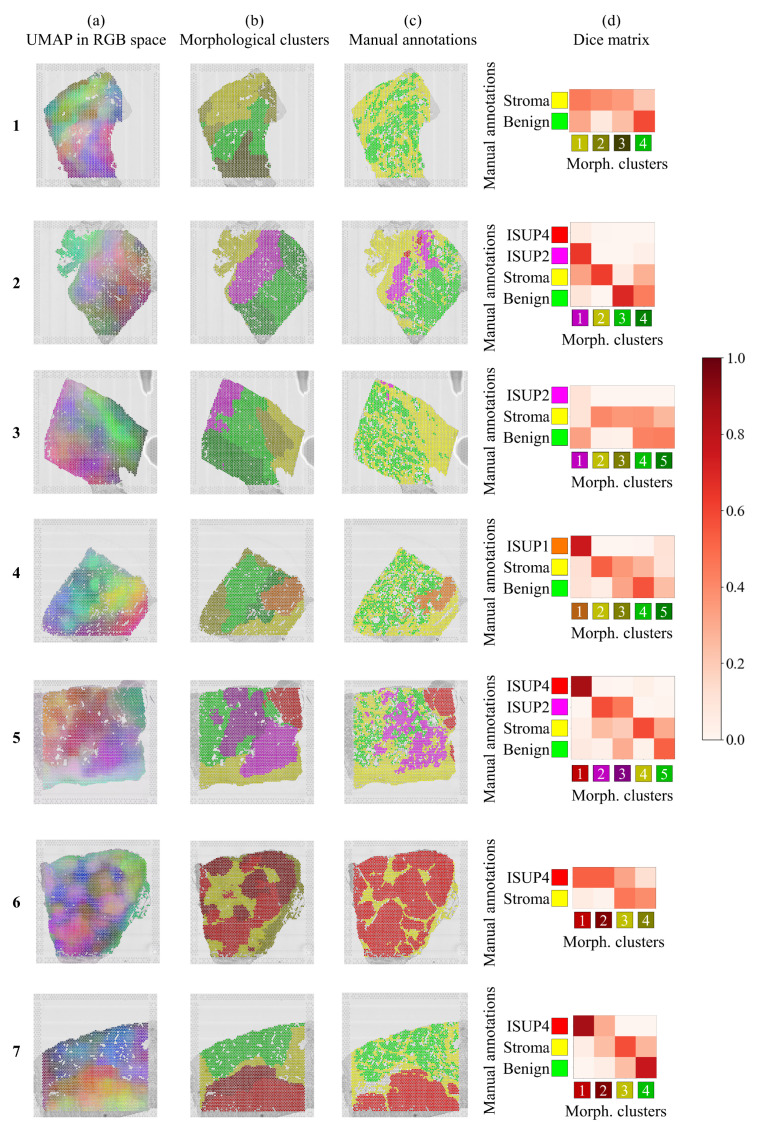
(**a**) Tissue heterogeneity is visualized by reducing the morphological feature space to three dimensions and presenting the morphological descriptors as RGB color maps. In this representation, a given color does not indicate any particular tumor grade. (**b**) Gaussian Mixture clustering using ten morphological descriptors provide clusters, where colors were deliberately chosen to match manual annotations. (**c**) Manual annotations of tumor grades performed by the histopathologists. In white color, we see spots excluded by the histopathologists that were not automatically discarded as being outside of the tissue by the ST method. (**d**) Dice matrices quantifying overlap between (**b**,**c**), the colorbar indicating the Dice coefficient, with values close to one representing exact spatial overlap, while values close to zero represent no spatial overlap.

In order to aggregate all the networks’ descriptions, we again applied UMAP dimensionality reduction to reduce the 10×30=300 features to three dimensions (3D). The colours shown in the 3D scatter plot in Figure 2c correspond to the three features normalized and interpreted as RGB dimensions. We also mapped the colored spots back to the original image to serve as a visualization of the tissue heterogeneity. Similarities/differences in color means similarities/differences in morphology, but colors do not indicate a particular tumor grade. We obtained RGB color descriptions of heterogeneity in deep morphological features for all spots in the seven available tissue sections, as shown in Figure 3a.

The same tissue sections were also manually/visually graded by two independent pathologists, and their annotations are shown in Figure 3c.

The automatically obtained color maps of morphological heterogeneity in Figure 3a concur visually with the independent manual annotations in Figure 3c. In section 1 of the figure, we see that the maps even have a slightly different color for the equivocal benign and the benign regions. Again, in section 2, we see different colors for the ISUP2 and ISUP4, even if the latter makes up a very small portion. The same is observed in section 3, where ISUP2 is a very small region. In section 4, the ISUP1 region was adequately differentiated, while the mix of benign and stroma to its left has mixed colors. In section 5, we observe that the same tissue annotation ISUP2 contains more than one color, suggesting possible morphological within-region differences detected by the network. The maps in section 6 identify all the tissue heterogeneity despite, again, having the same ISUP4 annotations in slightly different colors. Finally, we see that the difficult region in section 7 also has a different color as compared to the ISUP4 region.

This suggests that the method defines morphologically coherent regions, described only by three deep morphological features, that are comparable to the consensus of two histopathologists’ annotations.

### 2.4. Cancer Sub-Regions Relate to Manual Annotations

We additionally wanted to perform clustering and therefore embedded the 10×30=300 features into 10 dimensions for unsupervised clustering using a Gaussian Mixture Model (Figure 2d). To set the number of clusters, we applied the Calinski–Harabasz method [17]. We also tested K-means and spectral clustering, and even though there were slight variations in the resulting clusters, the three clustering algorithms yielded virtually the same clusters, mainly explained by the richness of the feature space (Supplementary Figure S9).

Automated clustering by Gaussian Mixture clustering, as shown in Figure 3b, provides a quantitative approach to compare automated and visual annotations. Note that we deliberately chose cluster color shades to ease visual comparison. We again found the same concordance with the histopathologists’ annotations, with different regions being detected using ten unsupervised morphological features. The Dice matrices in Figure 3d provide a quantitative analysis of the unsupervised cluster definitions.

It is sometimes the case that the slight differences observable by the feature map representation were coarser when committing to a specific cluster. For instance, in section 1 of the figure, we see that the equivocal benign region was clustered with the adjacent stromal region. In section 2, the low number of clusters loses the differences between ISUP2 and ISUP4 cancer. Furthermore, a similar effect is observed for section 3, where the region associated with ISUP2 cancer becomes much larger. For section 4, we observe an additional benign region defined around the ISUP1 cancer. Interestingly, in section 5, the separation of the ISUP4 region into two clusters persists, as does the unsure region in section 7. In section 6, some of the regions visually graded as ISUP4 were missed.

### 2.5. Morphological Clusters Relate to Gene Expression Factors

In order to study whether our automatically obtained morphological clusters were genetically relevant, we analyzed whether they corresponded to different gene expression factor signatures. The factors were obtained from spatially-resolved gene expression, as described in [8,9]. Note that the factors only cover the parts of the tissue where gene expression was measured.

**Figure 4:**
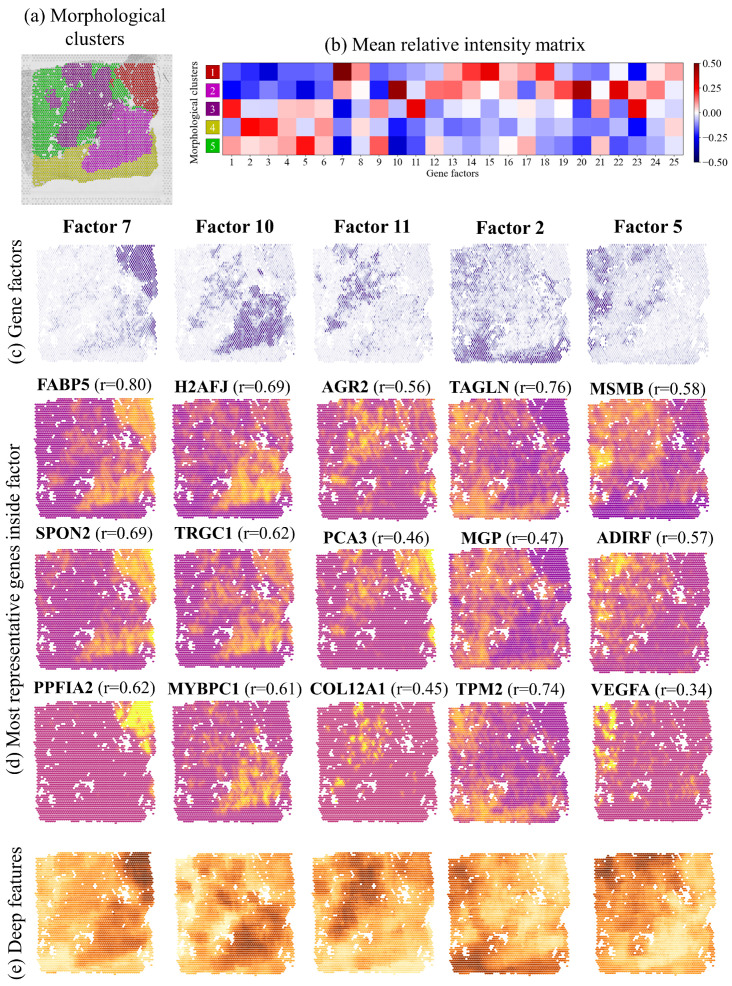
(**a**) Morphological clusters obtained by unsupervised clustering in ten-dimensional feature space for section 5. (**b**) Comparing gene expression factors with morphological clusters identifies the best-matching gene expression factor. (**c**) Visual presentation of the gene expression factors with the highest ratio for each cluster. (**d**) Genes with the largest number of reads for each selected factor (yellow and purple dots represent a large and small number of local reads, respectively). Values in parenthesis are standardized Pearson correlations of the genes with (**e**) the most significant feature of each of the morphological clusters 1–5.

Here, we refer to section 5 of the figure, where a region manually marked as containing ISUP2 cancer was automatically divided into two morphological sub-clusters, as shown in Figure 4a. We found that the sub-clusters are also represented by different gene expression factor signatures, as shown in Figure 4b. In this section, factor 10 is mainly contained in morphological cluster 2, corresponding to the lower half of the ISUP2 region defined by the histopathologists, while factor 11 is mainly contained in morphological cluster 3, corresponding to the upper half of the ISUP2 region, as shown in Figure 4c. While factor 10 genes are said to represent some combination of Gleason 4 patterns, factor 11 relates to monocytes. Furthermore, morphological cluster 1 correlates well with gene expression factor 7, related to Gleason 4 + 4 patterns, which was also annotated as ISUP4. Note that different clusters were created for the Gleason 4 patterns in ISUP2 and ISUP4, which ended up correlating with different gene expression factors. Morphological cluster 4 correlates well with factor 2, corresponding to the stroma annotation and factor. Cluster 5 correlates well with factor 5, which was identified as benign by the histopathologists and by factor analysis. Other tissue sections and corresponding comparisons to gene expression factors are presented in Figure S10 of the Supplementary Materials.

### 2.6. Spatial Expression of Individual Genes Predicted by Morphological Features

While carrying out inverse analysis, we further found that the expression of individual genes forming a factor (Figure 4d) is tightly correlated with the morphological features forming genetically relevant clusters (Figure 4e). The obtained Pearson correlation results (Figure 4d) are comparable or even higher than previous publications that trained networks specifically for ST prediction [14]. Similar representations for the rest of the sections are presented in Figure S10 of the Supplementary Materials. Again, we see that the spatial patterns described by unsupervised deep features correspond to the spatial expression of relevant genes.

Further interpretation of specific examples are presented in the Discussion.

### 2.7. Network Pre-Trained on Biological Images Find More Relevant Regions

We finally wanted to compare feature extraction using our network pre-trained on prostate needle biopsies to feature extraction using a network pre-trained on the ImageNet dataset (containing images of natural scenes). The results of feature extraction with a network pre-trained on ImageNet (while applying the same workflow as described above) are shown in Figure S11 of the Supplementary Materials. The non-medical network also finds visually similar regions, but they are much less defined and less biologically relevant, as compared to a random network from the ensemble.

This could be considered to define the lower sensitivity limit. Applying the network pre-trained on prostate needle biopsies on this dataset could be considered to be too specific and rigid, while a network pre-trained on ImageNet was not dedicated enough to find meaningful regions. The corresponding analogy would be asking a lay-person to grade the section: they would only define regions that were stained similarly, without the regions necessarily being relevant.

## 3. Discussion

The current study combines previous knowledge of morphological and genetic integration, overcoming important challenges suggested in the field. This flexible workflow allows one to extract morphological features in any desired dimensionality depending on the task at hand. To avoid misleading associations between very high-dimensional morphological and genetic information, both are first summarized using state of the art methods: UMAP dimensionality reduction and factor analysis, respectively.

Despite the models being blind to ST data, the correlation of morphological descriptors with gene expression were comparable with previous studies which trained a model specifically to predict individual gene expression [14]. Looking at the correlation, we could hypothesize that the genes that are more closely related to the deep morphological features have some role in cell and tissue changes related to cancer development.

For instance, in section 3 in Figure 3, although the ISUP2 annotation covers a very small part, the cluster including it is larger and genetically relevant. Specifically, the high expression of PDLIM5 in the whole cluster may indicate tumorgenesis and migration [18], something that the model detected only from morphology. Similarly, the region around the ISUP1 cancer in section 4 was annotated as benign but the model clustered it separately; it has high spatial expression of another migration-related gene TSPAN1 [19].

As we showed, in section 5, the ISUP2 region was separated into two morphological clusters. The lower region related to factor 10 that is composed of genes such as H2AFJ, shown to relate to tumor progression in other types of cancer [20] or TRGC1, which may be a marker for higher aggressiveness [21]. Conversely, the upper region associated with factor 11, that includes genes such as AGR2, which is over-expressed in low-grade cancer and PIN lesions [22]. Thus, although the tissue was consensually labeled as ISUP2, using our method, we were able to more finely grade the tissue.

The case of the region on top of the ISUP4 in section 7 is also worth mentioning. The histopathologists agreed that one could not confidently label the region as benign or cancerous. The network also created an additional cluster for this region which, through factor analysis, we found is related to the spatial expression of DEPDC1, which was found to be linked to tumor growth and cell proliferation in prostate cancer [23].

This creates the possibility to automatically annotate tissue slides combining morphological and transcriptomic information. Additionally, this workflow can be used for annotation by first describing the regions and then exploring the spatially resolved gene expression inside. The ST techniques alone are not directly mappable to the morphology [8], and there can be too many gene factors to easily choose which ones are relevant for every particular case. However, first defining the homogeneous regions and then analyzing the ST spots inside would integrate morphological and genetic information.

In a clinical setting, this approach could be used to help histopathologists annotate tumor samples by looking at the defined unsupervised regions, reducing the time needed for manual annotations, especially in the assessment of boundaries. The expert histopathologist intra-observer variability in grading prostate cancer is well known [16]. With this approach, we first find the regions and later assign labels, including the human in the loop [24].

Similar approaches can be used for other types of cancer. One could use challenge-winner networks in biology, extract their penultimate layer and use them as good surrogates of morphological features, instead of the current trend of using generalist pre-trained networks or networks trained on own data [25]. We further proved that the features are also closely correlated with gene expression, which opens the door to the integration spatial and genetic data using the most powerful AI techniques for each cancer.

Future work could also seek to relate the morphological features extracted by the model to the presence of specific cell populations, without the need to retrain with single cell data. Recent work on ST proposes that transcriptomic regions can be mapped to distinct cell types [26,27]. Thus, it would be interesting to continue studying the overlap between morphological descriptors extracted from H&E slides and both ST and single-cell sequencing.

However, in order to validate this kind of bold idea, some limitations should be overcome. First, a limited number of slides from a single patient was used in this study, due to the cost of ST. However, the sections were chosen so as to include different combinations of benign, stroma, and low- and high-grade cancer tissue.

Additionally, staining differences across slides and normalization is still challenging in automated histopathology [28]. We therefore applied the UMAP dimensionality reduction on a per section basis so the staining differences did not affect the final morphological clustering. It is important to note that there is always an inherent variability in histopathologist’s annotations. We only used the manual annotations for sanity checking to verify the morphological validity of our automatically obtained feature clusters.

Finally, the tissue patch size was selected to match that of the network we used for feature extraction, and was large compared to the ST spots. A smaller patch size would not have captured as much of the context, and it should be noted also that the histopathologists had access to the whole tissue view at visual grading. The drawback of the large patch size is that it produces a smoothing effect.

To conclude, we presented a workflow to repurpose a state-of-the-art AI system in order to define morphologically and genetically relevant sub-regions and find the associations between them. This continues to prove the close relationship between both levels of heterogeneity in prostate cancer and also supports the use of networks pre-trained on histopathology data for tumor grading in other applications. This is a new way to automatically detect prostate cancer without the need for direct visual inspection and, by also detecting genetic characteristics, raises the possibility that AI approaches such as this could differentiate cancer that needs treatment from indolent disease that does not.

## 4. Materials and Methods

### 4.1. Prostatectomy Sections with Spatially-Resolved Gene Expression

The H&E sections, together with the Visium ST data, were obtained upon petition and described in detail by Erickson et al. [9].

Annotations with few spots were merged with other annotations. Nerve, fat, vessel, and inflammation, which made up very few of the annotated spots, were merged with stroma. Section 1 contained a region that the histopathologists considered equivocal benign, shown in Figure S1; in Figure 3c, we relabeled it as benign. We also relabeled small regions with pre-malignant prostatic intraepithelial neoplasia (PIN) in sections 2 and 3 as benign, as shown in Figures S2 and S3. Finally, in section 7 (Figure S7), there is an excluded region in white on top of the ISUP4 cancer. This region was flagged by both histopathologists, as they were unable to classify it as benign or malignant with enough confidence. All annotations are included in Figures S1–S7 of the Supplementary Materials, together with the number of spots per annotation.

### 4.2. Image Acquisition and Registration

The slides were digitized using a Hamamatsu C9600-12 scanner with NDP.scan v.2.5.86 software (Hamamatsu Photonics, Hamamatsu, Japan). The pixel size at full resolution (40×) was 0.25 μm. The resulting images were stored at 8 bits for each RGB channel in TIFF format.

The ST coordinates (x,y), however, were located on compressed JPEG images. Thus, we registered the two datasets to obtain the coordinates in the full resolution $\left(x^{\prime },y^{\prime }\right)$. Using four fiducial points around each section, we applied analytical least squares to solve a similarity transform with four degrees of freedom consisting of scaling (*s*), rotation (*r*) and translation (*t*):

$$\left(\begin{array}{c} x^{\prime }\\ y^{\prime }\\ 1 \end{array}\right)=\left[\begin{array}{ccc} s\cdot r_{11} & s\cdot r_{12} & t_{x}\\ s\cdot r_{21} & s\cdot r_{22} & t_{y}\\ 0 & 0 & 1 \end{array}\right]\cdot \left(\begin{array}{c} x\\ y\\ 1 \end{array}\right)$$

### 4.3. Deep Morphological Feature Extraction

We used the ensemble of deep convolutional networks presented by Ström et al. [16]. It is an ensemble of 30 Inception V3 [29] networks trained to classify in four classes: benign, and Gleason grades 3, 4, and 5. The training and evaluation was performed on more than 7000 prostate needle biopsy cores, achieving clinically acceptable accuracy.

However, for our study, we needed to repurpose the system to be able to apply it to the prostatectomy slides and define the sub-regions. Using the ST spots as the center, we extracted 598×598 pixels patches at 10× resolution, roughly 540×540 μm, as the network was designed to receive. Conveniently, the Visium ST technique also contains information on whether the point is inside tissue or not. This resulted in 22,267 H&E patches, each associated with the spatial expression for tens of thousands of genes.

In order to obtain the morphological information for each of them, we fed the patches into the ensemble and extracted the 2048 penultimate layer activations for each of the 30 models, yielding a total of 2048×30 descriptors per patch. Then, uniform manifold approximation and projection (UMAP) for dimensionality reduction was applied to each model separately to embed each model into 10-dimensional spaces, obtaining 10×30 descriptors per patch.

### 4.4. Visualization and Clustering

At this point, we wanted to reduce the 10×30=300 morphological descriptors per spot to more manageable numbers.

On the one hand, for visualization, we used UMAP to reduce the dimensionality from 300 to 3 morphological descriptors per spot. This allows one to easily represent the ST spots of a section in a three-dimensional space. Additionally, assigning red, green, and blue (RGB) intensity values to each dimension, we could represent a color map on top of the original section, to observe the captured heterogeneity.

On the other hand, for clustering, we used UMAP to reduce the dimensionality from 300 to 10 morphological descriptors per spot. We experimentally confirmed that clustering with more than 10 features per spot did not yield novel morphological features in each section, suggesting that the features were starting to become redundant. That is, reducing the dimensionality to more than 10 features produced spatial maps which were linear combinations one of the other.

The 10 features per spot were clustered using Gaussian Mixture clustering. To set the number of clusters in every section, the Calinski–Harabasz [17] score used was between 4, which the original number of classes in the model, and 8, twice as many. Then, a threshold was set at 90% of the maximum value and the highest number of clusters above it was set. This was carried out to ensure that the network tended to find sub-regions.

### 4.5. Comparing Morphological Sub-Regions to Manual Annotations

To numerically compare the spatial overlap of the obtained clusters with the histopathologists manual annotations we constructed a Dice index matrix for each annotation $A_{i}$ and cluster $C_{k}$:

$$D\left(A_{i},C_{k}\right)=\frac{2\left|A_{i}\cap C_{k}\right|}{\left|A_{i}\right|+\left|C_{k}\right|}$$

### 4.6. Gene Factor Analysis and Morphological Sub-Regions

To check whether the clusters obtained by unsupervised clustering of morphological features were also genetically meaningful, we performed a joint analysis with gene factors. We used the gene factors obtained from the gene factor analysis technique [8,9], which summarizes the spatial expression of tens of thousands of genes into 25 gene factors. We constructed a relative mean intensity matrix by calculating the difference between the mean factor intensity, I(x), inside and outside the region described by the cluster, $C_{k}$, for each factor $F_{j}$ and dividing by the sum of all the mean factor intensities:

$$R_{F_{j},C_{k}}=\frac{I\left(F_{j}\left[C=k\right]\right)-I\left(F_{j}\left[C\neq k\right]\right)}{I\left(F_{j}\left[C=k\right]\right)+I\left(F_{j}\left[C\neq k\right]\right)}$$

### 4.7. Gene Expression Correlation

For each of the resulting factors, we selected the top genes with the most similar spatial pattern to the factor and calculated the Pearson correlation between the standardized gene expression and the morphological feature that contributed most for the corresponding cluster, as detailed in Figure S12.

### 4.8. Comparison with Other Clustering Algorithms and ImageNet Pre-Trained Network

We compared the results of clustering the 10 final UMAP components for section 5 into five clusters using Gaussian Mixture, K-means and spectral clustering (Figure S9). For comparability, we used default values in the scikit-learn package [30] for all of them. We also repeated the workflow in Figure 2 using a network pre-trained on the ImageNet dataset to evaluate whether the additional training was useful to find sub-regions in the section. We used exactly the same Inception V3 network architecture [29] and chose one of the 30 ensemble networks for comparison. Gaussian Mixture clustering with the default configuration was used again for both.

### 4.9. Software

All analyses were made on a local cluster using Python v. 3.6.9. We used Openslide v. 3.4.1 [31] to access and divide the images into patches, and scikit-image v. 0.17.2 package [32] for image processing. The network to extract morphological features was loaded via TensorFlow v. 2.5.0 and dimensionality reduction [33] was performed with the Python implementation for UMAP v. 0.5. Scikit-learn v. 0.23.2 package [30] was used for clustering. Finally, multi-resolution visualizations were created using TissUUmaps [34] (https://tissuumaps.research.it.uu.se/).

## Data Availability

The data and materials are available from the references and upon request.

## Supplementary Materials

The following are available online at https://www.mdpi.com/article/10.3390/cancers13194837/s1, Figure S1. Complete annotations for section 1. Benign* = equivocal benign. Figure S2. Complete annotations for section 2, PIN = prostatic intraepithelial neoplasia. Figure S3. Complete annotations for section 3, PIN = prostatic intraepithelial neoplasia. Figure S4. Complete annotations for section 4, PIN = prostatic intraepithelial neoplasia. Figure S5. Complete annotations for section 5. Figure S6. Complete annotations for section 6. Figure S7. Complete annotations for section 7. Figure S8. More than 10 features results in redundant feature maps. If the models’ 30 × 10 = 300 features are reduced to 20 dimensions, many of the maps are redundant; some of them are negatives or linear combinations of others, meaning that they would not contribute to new clusters, but rather confirm already found clusters. Figure S9. Comparison of clustering 10 UMAP components into 5 clusters using different algorithms for section 5. Figure S10. Comparison of morphology and gene factors in Section 1–4, 6 and 7. Figure S11. Comparison with ImageNet pre-trained network. Figure S12. Scatter plots of the normalized expression for all genes appearing in Figure 4 and their corresponding morphological features.

## Abbreviations

The following abbreviations are used in this manuscript:

H&E	Hematoxylin and eosin
ST	Spatial transcriptomics
ISUP	International Society of Urological Pathology
CNN	Convolutional neural network
AI	Artificial intelligence
RGB	Red, green, and blue
FDR	False discovery rate
