# Supplementary material for: Morphological Features Extracted by AI Associated with Spatial Transcriptomics in Prostate Cancer
Source: Cancers (Basel). 2021 Sep 28;13(19):4837. doi: 10.3390/cancers13194837 (PMC8507756; doi:10.3390/cancers13194837)
Supplement: Supplementary file 1 [file cancers-13-04837-s001.zip › cancers-1320051-supplementary.pdf]

Supplementary Materials: Morphological Features Extracted by AI Associate with Spatial Transcriptomics

Eduard Chelebian, Christophe Avenel, Kimmo Kartasalo, Maja Marklund, Anna Tanoglidi, Tuomas Mirtti, Richard Colling, Andrew Erickson, Alastair D. Lamb, Joakim Lundeberg and Carolina Wählby

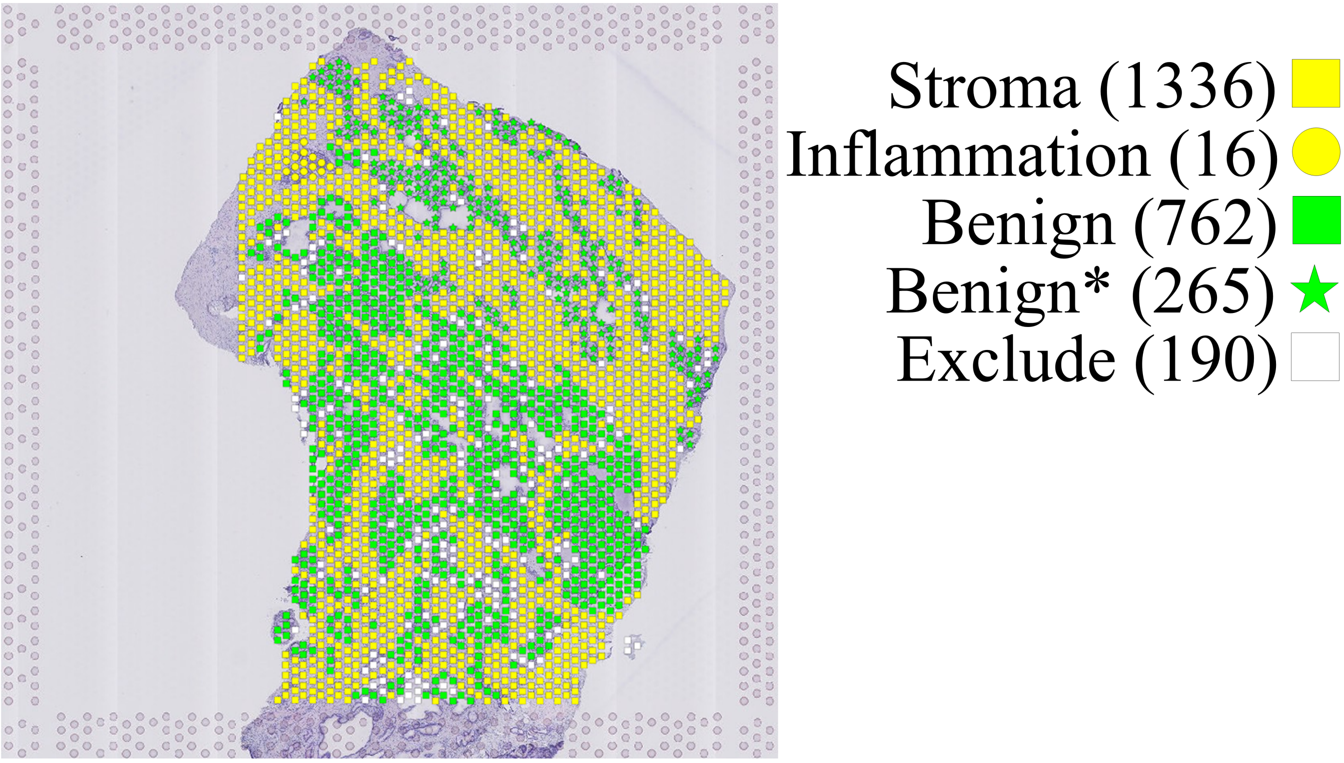

Figure S1. Complete annotations for section 1, from [1]. Benign* = equivocal benign.

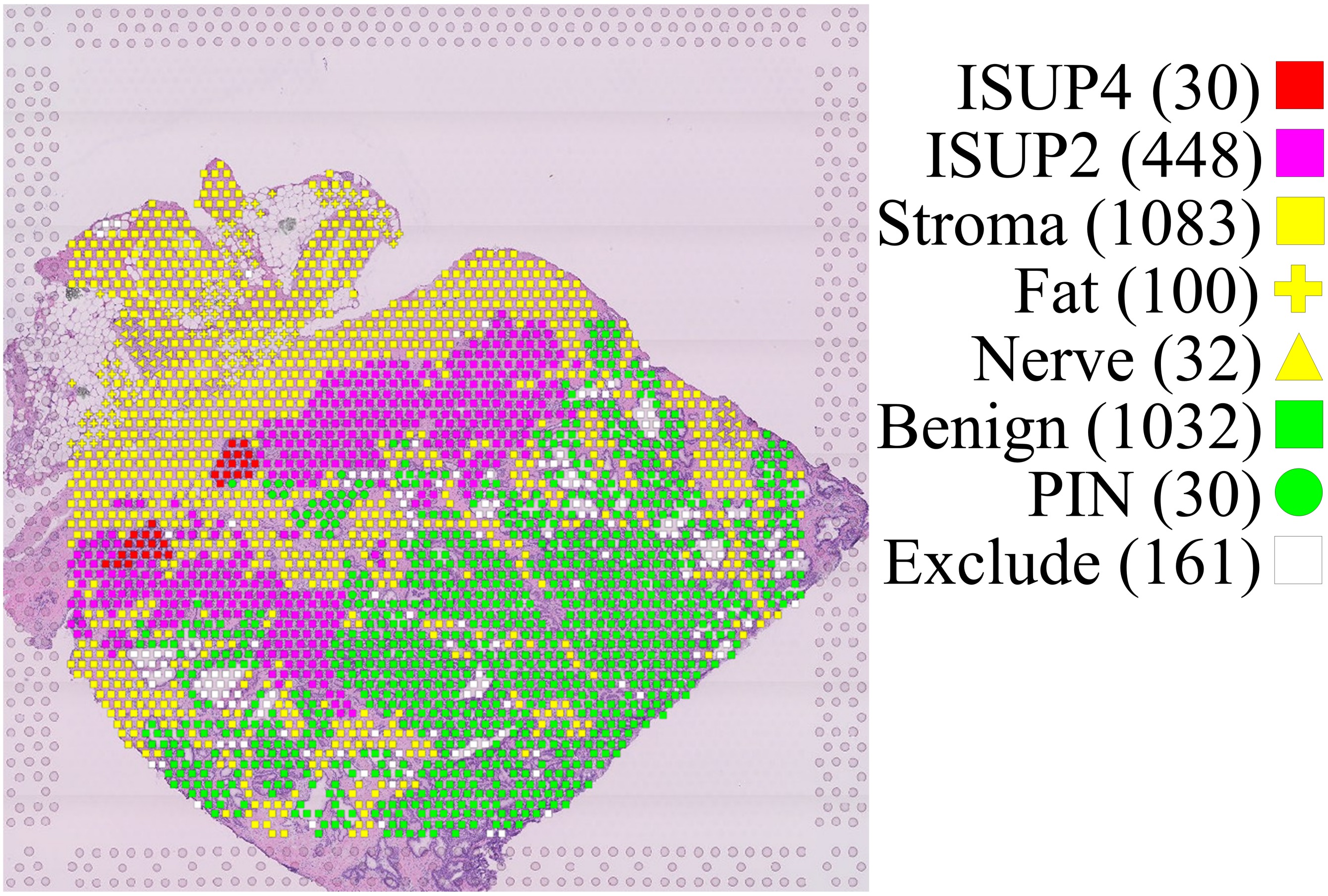

Figure S2. Complete annotations for section 2, from [1]. PIN = prostatic intraepithelial neoplasia.

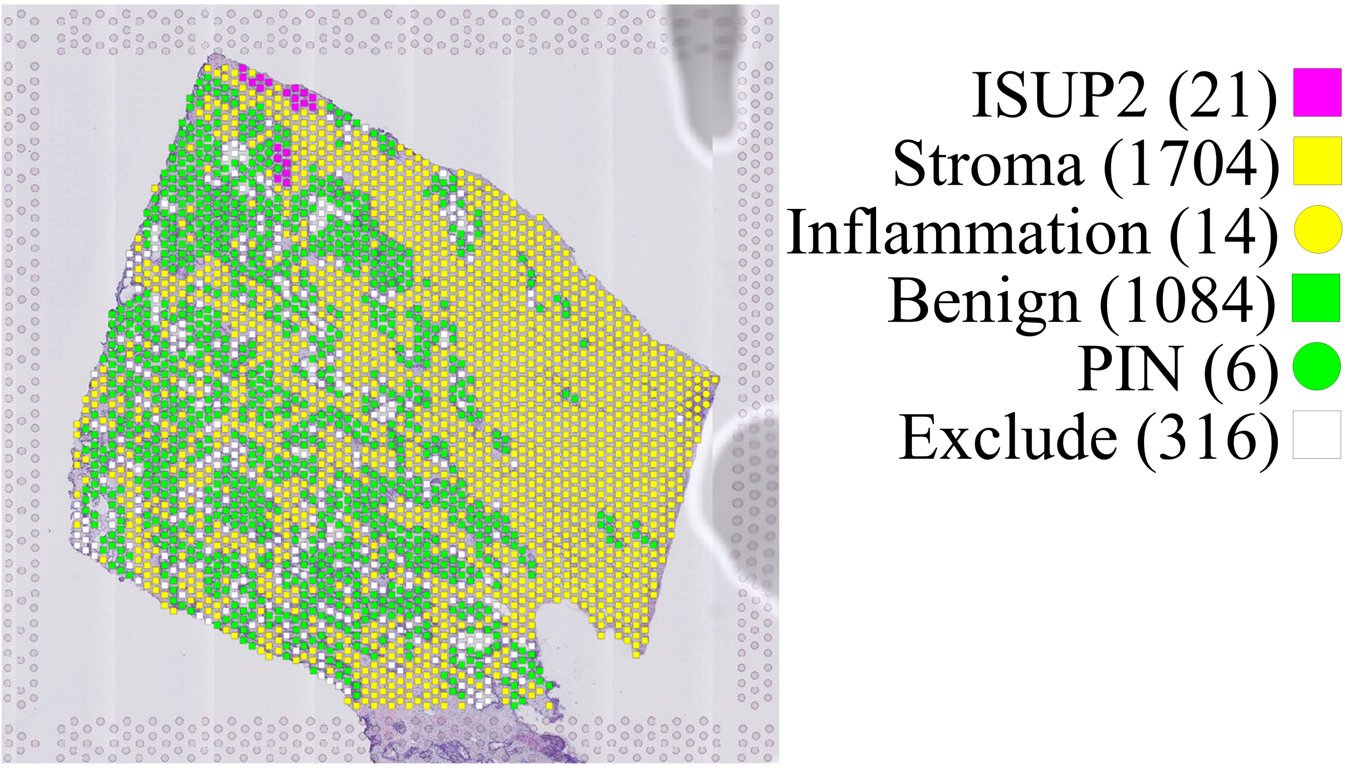

Figure S3. Complete annotations for section 3, from [1]. PIN = prostatic intraepithelial neoplasia.

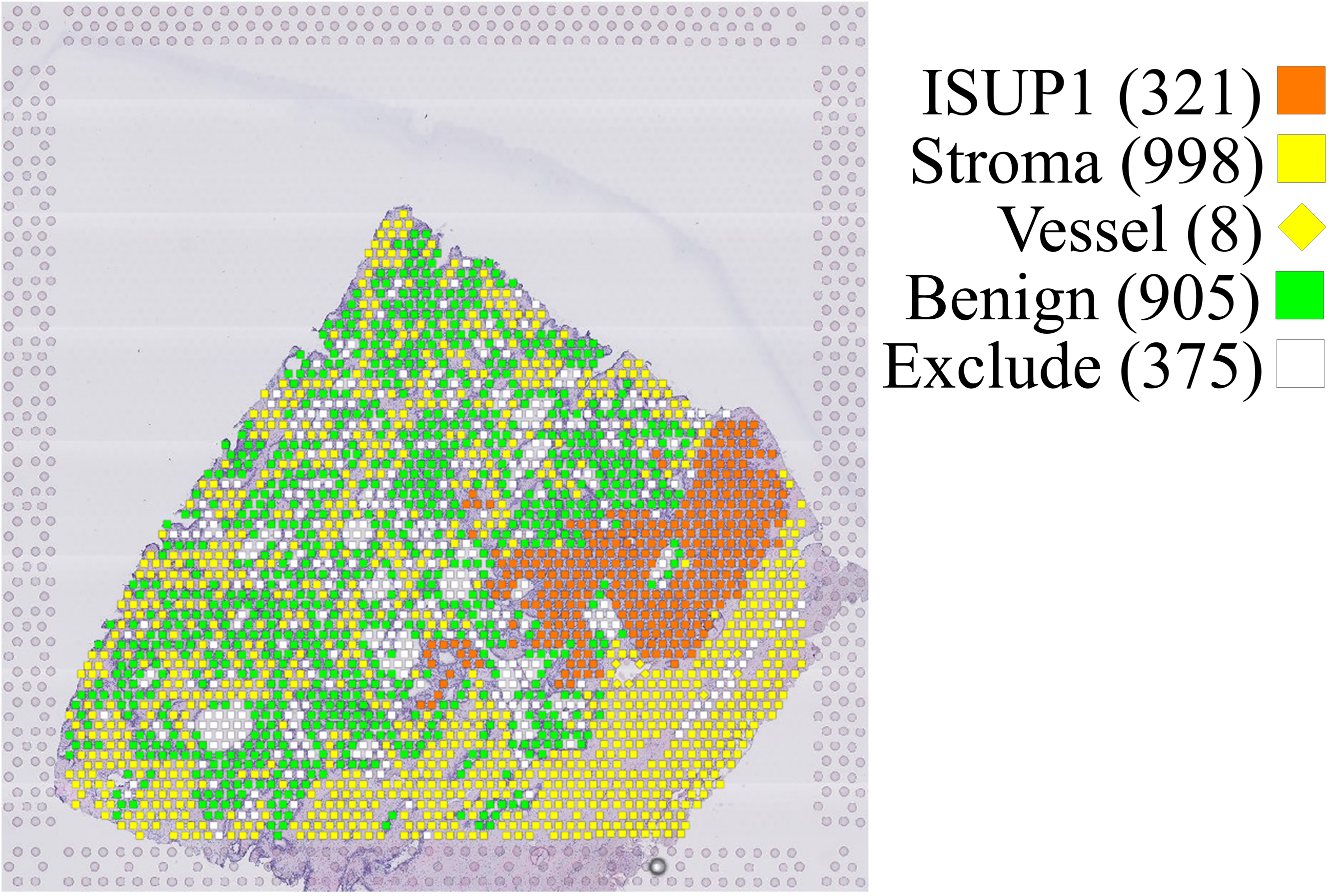

Figure S4. Complete annotations for section 4, from [1]. PIN = prostatic intraepithelial neoplasia.

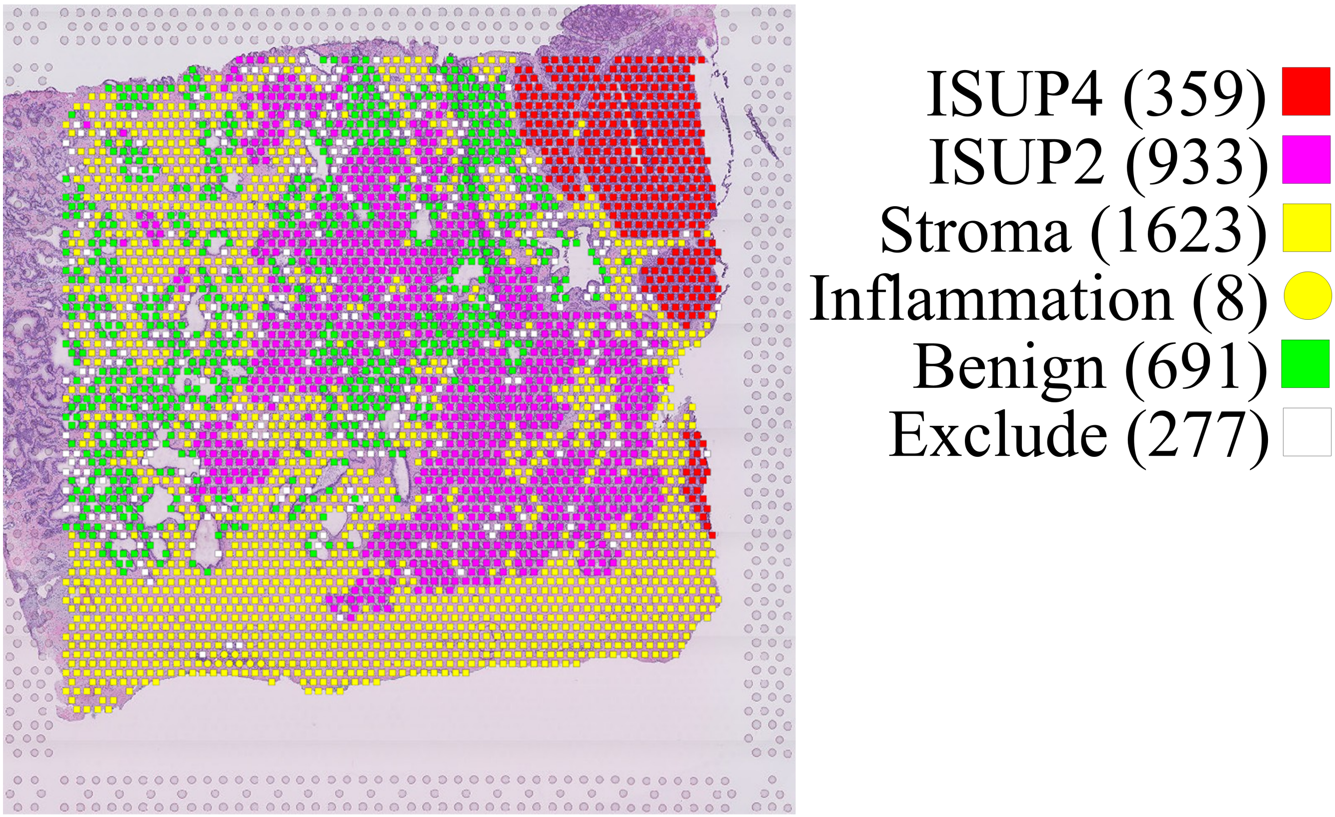

Figure S5. Complete annotations for section 5, from [1].

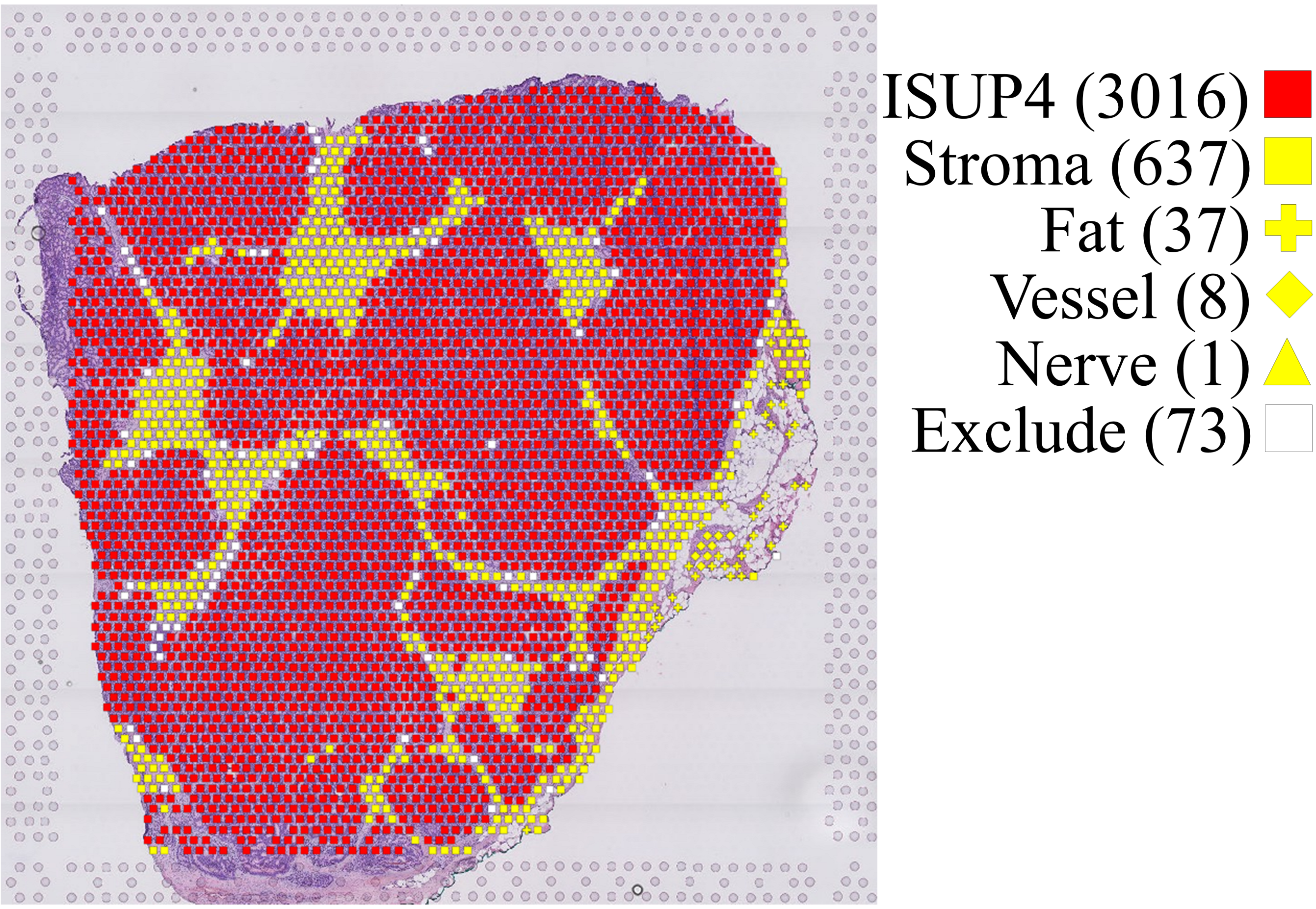

Figure S6. Complete annotations for section 6, from [1].

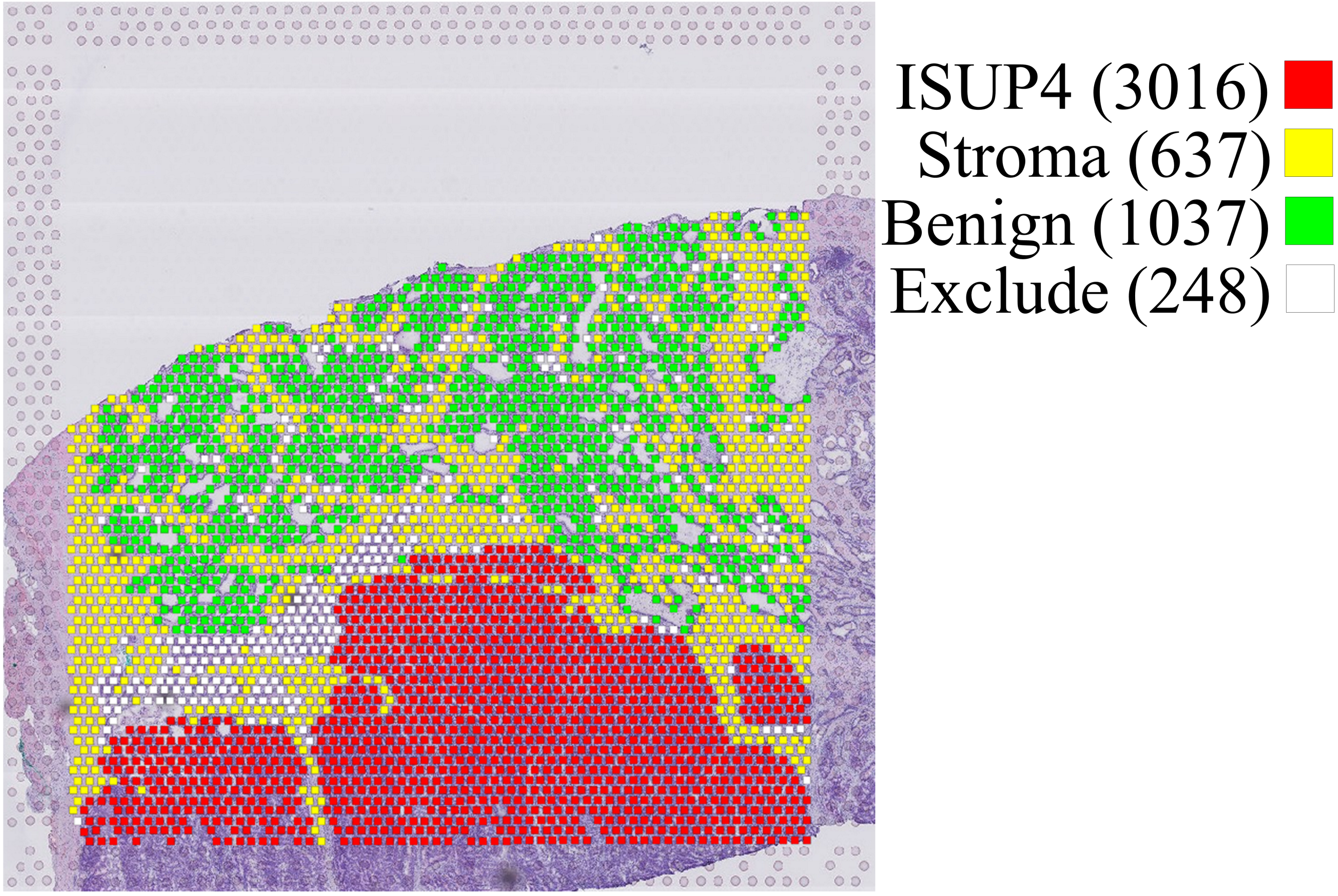

Figure S7. Complete annotations for section 7, from [1]. The excluded region on top of the ISUP4 tissue was labelled as *transition zone* because the histopathologists could not determine confidently whether it was benign or cancerous.

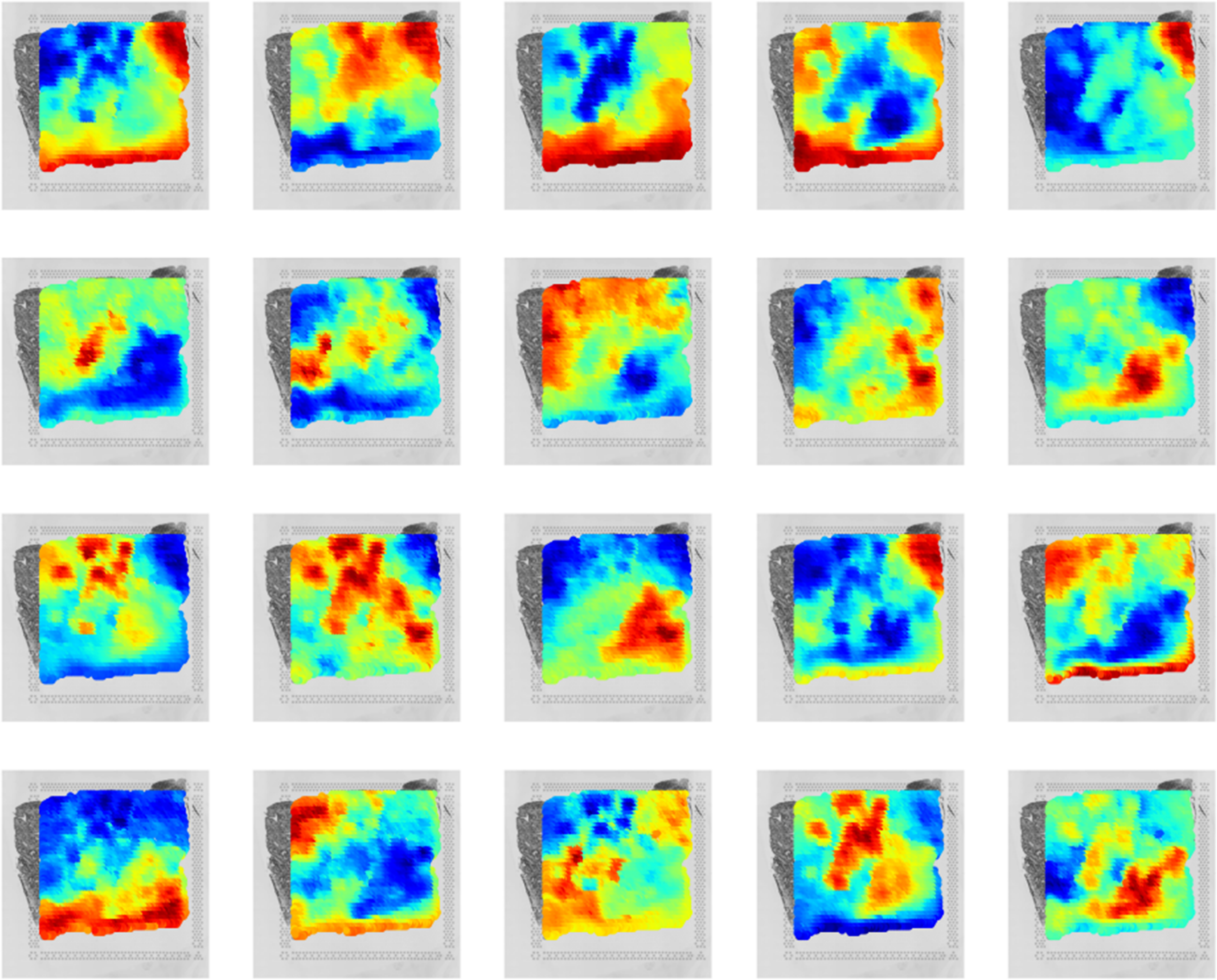

Figure S8. More than 10 features results in redundant feature maps. If the models' 30x10=300 features are reduced to 20 dimensions, many of the maps are redundant; some of them are negatives or linear combinations of others, meaning that they would not contribute to new clusters, but rather confirm already found clusters.

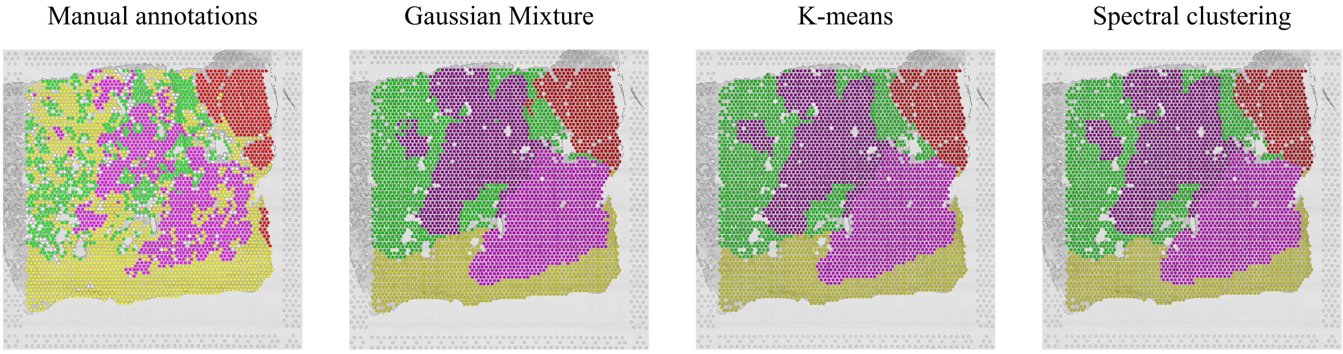

Figure S9. Comparison of clustering 10 UMAP components into 5 clusters using different algorithms for section 5.

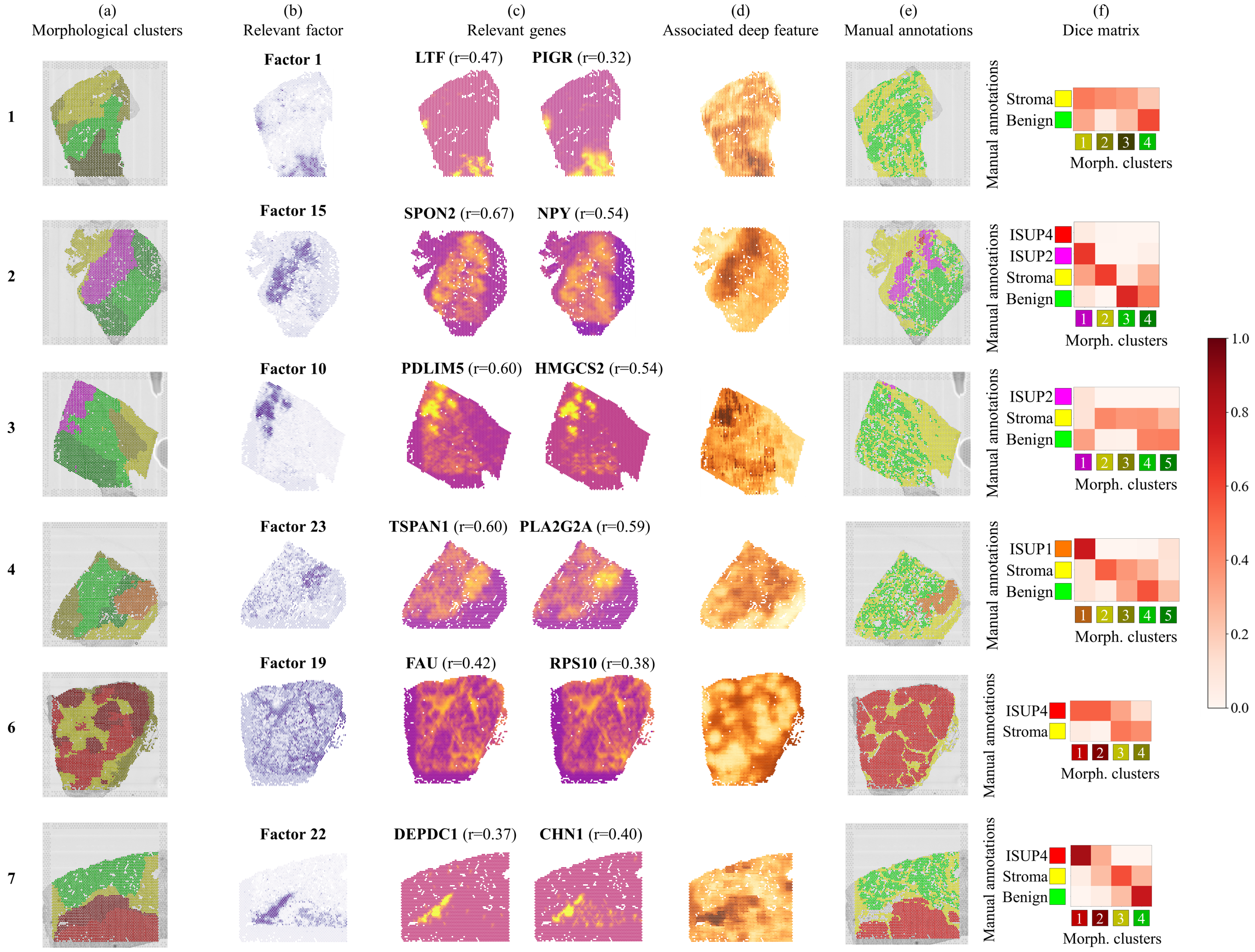

Figure S10. Comparison of morphology and gene factors in sections 1-4, 6 and 7. We focus on the clusters that correspond to regions not defined by the histopathologists. Factor 1 for section 1 does not clearly correspond to a specific annotation, instead it is a combination of stroma and benign tissue that the automatic clustering grouped in cluster 3. The interpretation of the factor confirms that it also represents monocyte presence. In section 2, cluster 1 merged ISUP2 and ISUP4 regions, but the factor analysis reveals that it is very similar to factor 15, in a region which is said to broadly represent some combination of Gleason pattern 4. Similarly, cluster 1 in section 3 is also genetically meaningful even if it does not match the histopathologists' annotation, as factor 10 is also highly expressed there. Interestingly, cluster 5 which was around the ISUP1 cancer has high relative intensity for factor 23, which indicates that indeed there is a different gene expression in this region as compared with the rest of the benign tissue. In section 6, cluster 3 contains a high portion of factor 19 which contains stromal genes. Finally, cluster 2 that corresponds to the part excluded by the histopathologists, and factor 22, have a similar spatial distribution. The region is said to be related to Gleason pattern 4.

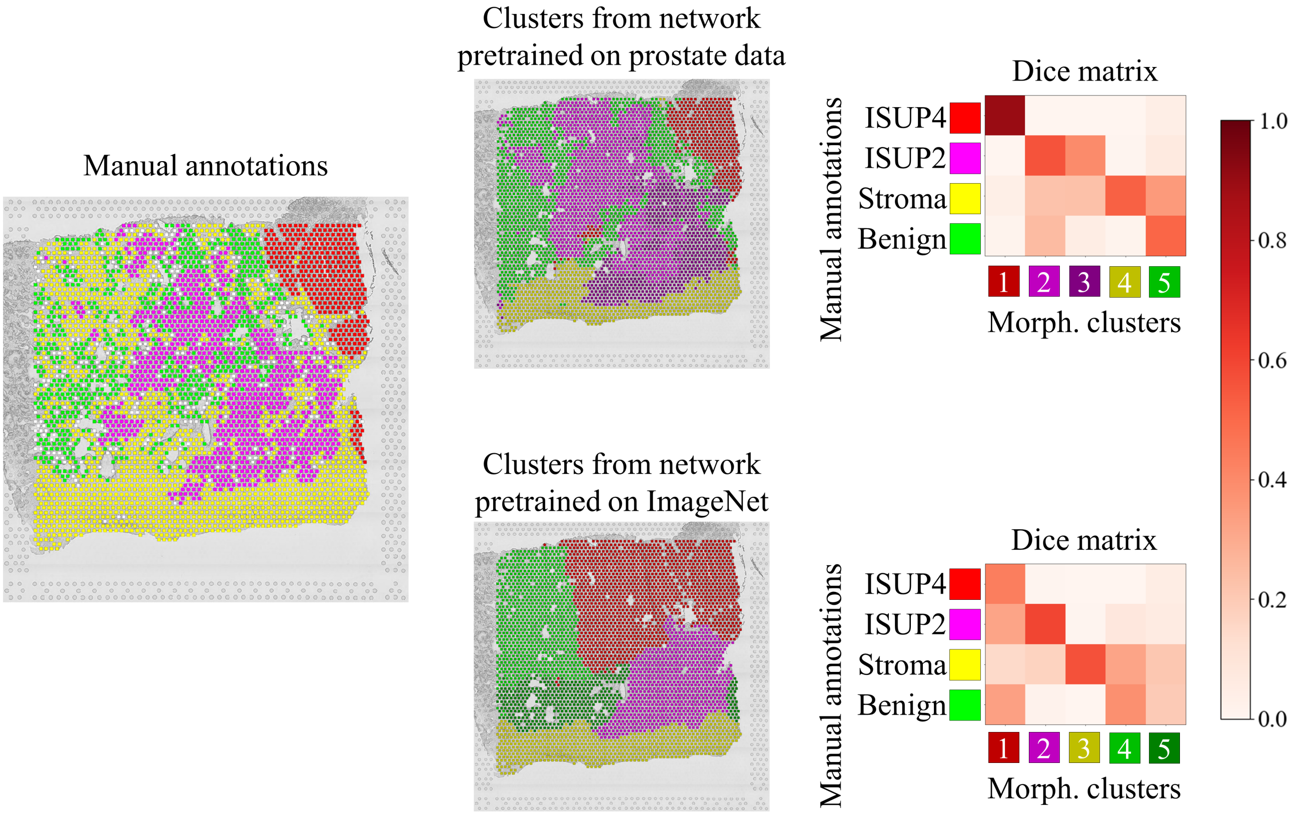

Figure S11. Comparison with ImageNet pre-trained network.

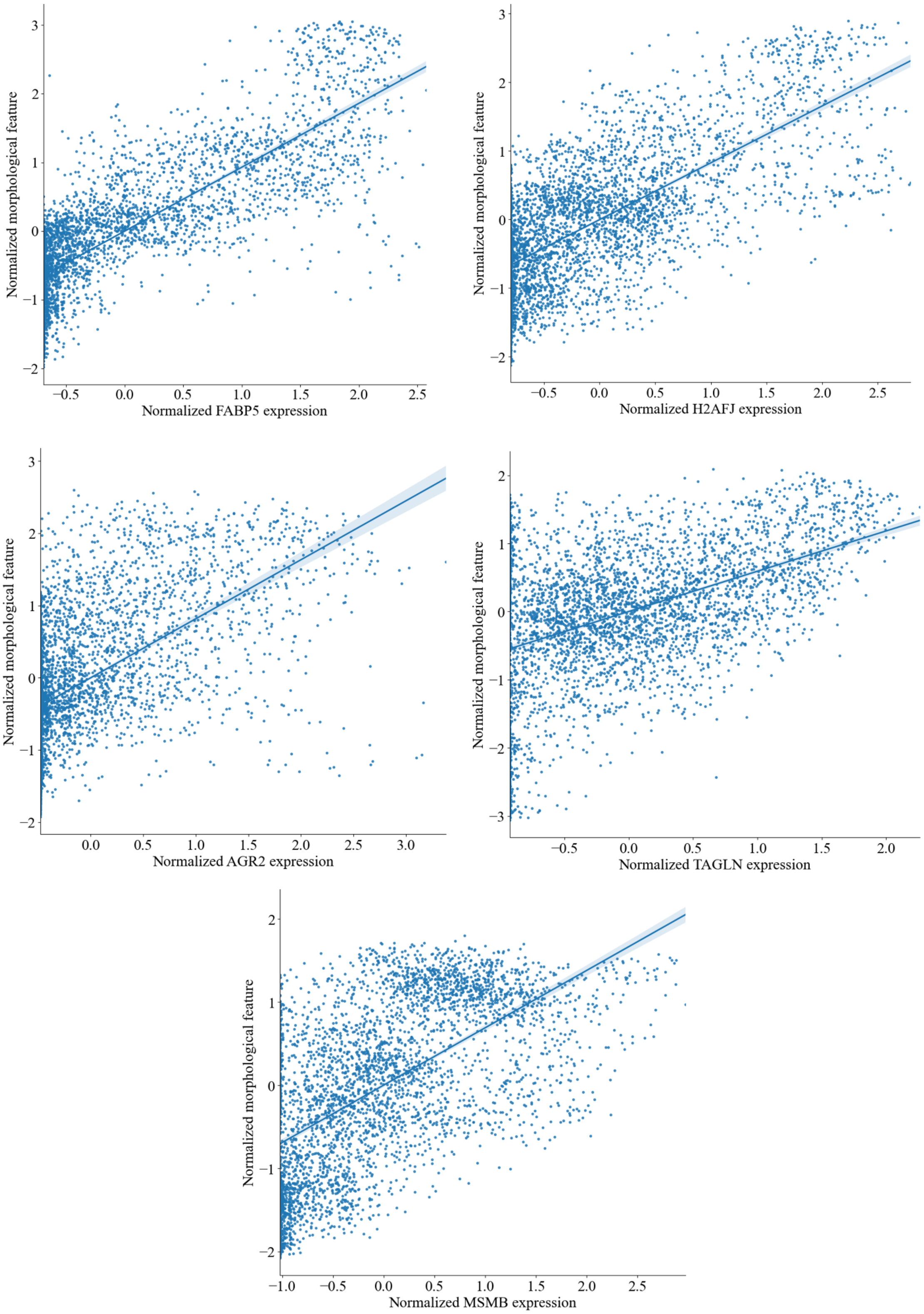

Figure S12. Scatter plots of the normalized expression for all genes appearing in Figure 4 and their corresponding morphological features. Pearson correlation coefficient is sensitive to outliers, and we cannot expect our variables to be normally distributed. These plots verify the absence of outliers that could otherwise have a large influence on the Pearson's r. Typically, when we Z-normalize, abs(value)>3 can be considered anomalies. In our data there is no point that deviated more than 3 standard deviations.

References

1. Erickson et al. The spatial landscape of clonal somatic mutations in benign and malignant tissue. *BioRxiv* **2021**.
